# Association of Plasma C1q/TNF-Related Protein 3 (CTRP3) in Patients with Atrial Fibrillation

**DOI:** 10.1155/2020/8873152

**Published:** 2020-12-24

**Authors:** Liwen Chen, Shuwang Liu, Wei Xu, Yuan Zhang, Jin Bai, Lei Li, Ming Cui, Lijie Sun

**Affiliations:** ^1^Department of Cardiology and Institute of Vascular Medicine, Peking University Third Hospital, 49 Huayuan-Bei Road, Haidian District, Beijing 100191, China; ^2^Key Laboratory of Cardiovascular Molecular Biology and Regulatory Peptides, Ministry of Health, 49 Huayuan-Bei Road, Haidian District, Beijing 100191, China; ^3^Key Laboratory of Molecular Cardiovascular Science, Ministry of Education, 49 Huayuan-Bei Road, Haidian District, Beijing 100191, China; ^4^Beijing Key Laboratory of Cardiovascular Receptors Research, Beijing 100191, China

## Abstract

Atrial fibrillation (AF) is a highly prevalent cardiac arrhythmia characterized by atrial remodeling. Complement C1q tumor necrosis factor-related protein 3 (CTRP3) is one of the adipokines associated with obesity, diabetes, and coronary heart disease. The association between plasma CTRP3 levels and AF is uncertain. The aim of this study was to investigate whether plasma CTRP3 concentrations were correlated with AF. Our study included 75 AF patients who underwent catheter ablation at our hospital and 47 sinus rhythm patients to determine the difference in plasma CTRP3 concentrations. Blood samples before the ablation were collected, and ELISA was used to measure the concentrations of CTRP3. Plasma CTRP3 concentrations were significantly lower in AF patients compared with control group (366.9 ± 105.2 ng/ml vs. 429.1 ± 100.1 ng/ml, *p* = 0.002). In subgroup studies, patients with persistent AF had lower plasma CTRP3 concentrations than those with paroxysmal AF (328.3 ± 83.3 ng/ml vs. 380.0 ± 109.2 ng/ml, *p* = 0.037). The concentrations of plasma CTRP3 in the recurrence group after radiofrequency catheter ablation of AF were lower than those in the nonrecurrence group (337.9 ± 77.3 ng/ml vs. 386.6 ± 108.1 ng/ml, *p* = 0.045). Multivariate regression analysis revealed the independent correlation between plasma CTRP3 level and AF. Plasma CTRP3 concentrations were correlated with the presence of AF and AF recurrence.

## 1. Introduction

Atrial fibrillation (AF) is one of the most common arrhythmia in clinical practice. Disability and morbidity due to AF remain high [1]. Over the past decades, research has identified a multitude of pathophysiological processes contributing to the initiation, maintenance, and progression of AF. However, many aspects of AF pathophysiology have not yet been fully elucidated. Current studies suggest that atrial remodeling [2], inflammatory factors [3], oxidative stress [4], and the effects of the autonomic nervous system [5] are mechanisms related to AF development. Besides, the treatment of atrial fibrillation is also essential. Nowadays, catheter-based ablation technologies which have made the procedure safer, more comfortable to perform, and more effective after a single attempt have greatly improved the outcomes, but AF recurrence is still common [1, 6].

In recent years, the relationship between adipokines and AF has been well recognized. Adipokines are involved in the inflammatory response, immune response, insulin resistance, obesity, metabolic syndrome, etc. [7]. A large number of studies have shown that plasma classic adipokines such as adiponectin, resistin, and leptin play a role in AF through inflammatory factors and oxidative stress [8, 9]. New adipokines including visfatin, chemerin, and complement C1q tumor necrosis factor-related protein 3 (CTRP3) are another cluster of active endocrine cytokines secreted by adipocytes [10]. CTRP3, a newly discovered adipokine with sequence homology with adiponectin, is involved in regulating energy metabolism and cardiovascular function [11]. CTRP3 has been found to regulate endocrine secretion, glycolipid metabolism, and immune and inflammatory responses and regulate mitochondrial biosynthesis, angiogenesis, vascular calcification, and ventricular remodeling [12–14]. Therefore, we hypothesized that CTRP3 might mediate the mechanism of AF as well. While to date, no study examined plasma CTRP3 concentrations in patients with atrial remodeling and AF. Therefore, we conducted a case-control study to determine plasma CTRP3 concentrations in patients with AF and explored associated factors and the potential clinical significance of plasma CTRP3.

## 2. Material and Methods

### 2.1. Patients

A case-control study was conducted in patients with AF who were admitted to the Department of Cardiology, Peking University Third Hospital, between January 2018 and January 2019 and patients with sinus rhythm as control. AF patients who were ready for radiofrequency ablation (aged ≥ 18 years) were recruited in the AF group. The diagnosis criteria of AF were using the 2014 AHA/ACC/HRS Guideline for the Management of Patients with Atrial Fibrillation [6]. We excluded the patients with congestive heart failure (New York Heart Association (NYHA) class III or IV), dilated or hypertrophic cardiomyopathy, valvular heart disease, congenital heart disease, acute coronary syndrome, malignant tumor, malignant hematonosis, autoimmune disease, infection, severe liver disorders (AST > 50 U/l or ALT > 40 U/l), kidney disorders(creatinine > 115 *μ*mol/l), and acute phase of cerebrovascular disease and those who had surgery or trauma in the past 3 months. AF patients were further divided into two subgroups: paroxysmal AF group and persistent AF group. Paroxysmal AF was defined as AF episodes lasting less than seven days and can be terminated spontaneously. Persistent AF was defined as AF episodes lasting more than seven days or requiring termination with pharmacologic or direct current cardioversion [6]. For comparison, patients with sinus rhythm who were admitted during the same period were included in the control group.

### 2.2. Ethics

This study was approved by the Ethics Committee of the Peking University Third Hospital Medical Science Research.

### 2.3. Clinical Data Collection

The study included demographic data and vital signs on admission and calculated body mass index (BMI). Clinical medications were recorded, and information regarding concomitant diseases was collected.

### 2.4. Clinical Laboratory Indicators

Venous blood samples were collected within 24 hours of admission. The following clinical, biochemical markers were measured at the laboratories of Peking University Third Hospital: ultrasensitive C-reactive protein (US-CRP), creatinine (Cr), hemoglobin A1C (HbA1C), total cholesterol (TC), triglycerides (TG), low-density lipoprotein cholesterol (LDL-C), high-density lipoprotein cholesterol (HDL), and N-terminal probrain natriuretic peptide (NT-proBNP).

### 2.5. Echocardiography

Two-dimensional transthoracic echocardiography was performed using GE Vivid E9 as the same with a 3.5 MHz transducer. Transthoracic echocardiography was completed by experienced doctors on all subjects to evaluate the characteristics of their left atrial area (LAA), left atrial diameter (LAD), left atrial pressure (LAP), and left ventricular ejection fraction (LVEF) at the time of admission.

### 2.6. Follow-Up and AF Recurrence Assessment

All participants had regular follow-up visits with a phone, online interview, 12-lead electrocardiograph (ECG)/72-hour Holter, medical history, and clinical evaluation. The follow-up period was 12 months. Every symptomatic patient was referred to a new ECG. Recurrence of AF postablation is defined as a recurrence of AF/atrial flutter/atrial tachyarrhythmia of at least 30 s, documented by an ECG or device recording system after a blanking period of 3 months [15].

### 2.7. Measurement of CTRP3 Concentrations

Venous blood samples were collected from every patient while in a fasting state after 30-minute rest in the sitting position. Besides, venous blood was collected before radiofrequency ablation in AF patients. Samples were taken from the cubital vein into blood tubes and immediately stored on the ice at 4°C. Plasma samples were processed within 30 min after collection by centrifugation at 3000 g for 15 min at 4°C. To avoid repeated freeze-and-thaw cycles, each plasma sample was divided into 0.2 ml aliquots and frozen immediately at -80°C. Plasma CTRP3 concentrations were determined by enzyme-linked immune sorbent assay kits (Cloud-Clone Corp, Inc.).

### 2.8. Statistical Analysis

Normally distributed data were presented as means ± standard deviation, nonnormally distributed data were reported as medians with interquartile range, and categorical variables were expressed as numbers and percentages. Comparisons between groups for the normally distributed data were performed with unpaired *t*-tests. Nonparametric tests were undertaken for comparisons between groups of nonnormally distributed data. The chi-square test was used for comparisons of categorical variables. Multivariate analysis was carried out using the Binary logistic regression model. Correlation between the normally distributed data was calculated by the Pearson correlation test, and the correlation between the nonnormally distributed data were analyzed through the Spearman correlation test. All statistical analyses were computed in a commercially available statistical calculation program (SPSS 23.0, SPSS Inc., Chicago, IL). Figures were analyzed using GraphPad Prism (GraphPad Software Inc., San Diego, CA). Two-tailed *p* value < 0.05 was considered of statistical significance.

## 3. Results

### 3.1. Baseline Clinical Characteristics

75 AF patients and 47 patients with sinus rhythm were included in the study. Among the 75 AF patients, 56 of them were paroxysmal AF, and 19 were persistent AF. The average duration of the study population was 50 months. Clinical characteristics and laboratory findings at baseline were summarized in Table 1. The AF patients were older than the patients with sinus rhythm (62.5 ± 11.7 vs. 56.9 ± 11.4, *p* = 0.011), whereas there were no significant differences in sex or BMI between the two groups (*p* > 0.05). Anticoagulant drugs were more widely used in the AF population (21 vs. 0, *p* < 0.001). The proportion of current smokers and patients with concomitant diseases was similar between the two groups. At the same time, there is no difference in the use of antiplatelet agents, calcium channel blockers, beta-blockers, angiotensin-converting enzyme inhibitor/angiotensin receptor blockers, or statins observed between the two groups. Plasma NT-proBNP was higher in the AF patients (*p* < 0.001). No significant differences were found in other clinical indicators, including blood pressure, lipid profile, HbA1c, renal function, and US-CRP. As for echocardiographic indicators, LAA and LAD were higher in the AF patients, and LVEF was lower in AF patients.

### 3.2. Comparison of Plasma CTRP3 Concentrations between Patients with Sinus Rhythm and AF Patients, and between the Subgroups

**Figure 1:**
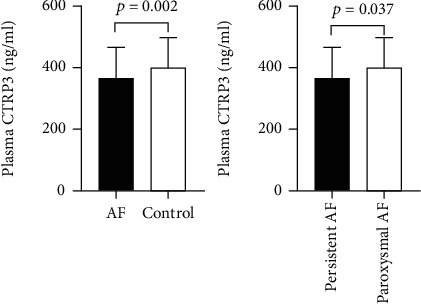
Comparison of plasma CTRP3 concentrations between patients with sinus rhythm and AF patients and between the subgroups.

**Figure 2:**
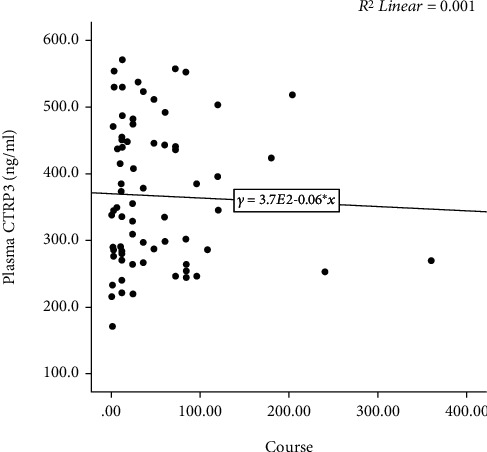
Relationship between plasma CTRP3 and AF course.

Plasma CTRP3 concentrations in the AF group were lower than that in the control group (366.9 ± 105.2 ng/ml vs. 429.1 ± 100.1 ng/ml, *p* = 0.002) (Figure 1). The characteristics of AF subgroups were shown in Table 2. Furthermore, we found that plasma CTRP3 concentrations were significantly different among the subgroups. It was higher in paroxysmal AF patients than that in persistent AF patients (380.0 ± 109.2 ng/ml vs. 328.3 ± 83.3 ng/ml, *p* = 0.037) (Figure 1). However, there was no significant correlation between plasma CTRP3 concentrations and the duration of AF (Figure 2).

When we compared the plasma CTRP3 concentrations in the AF patients according to the cardiac rhythm at admission, no significant difference was found between the patients with sinus rhythm (*n* = 40) and patients with AF (*n* = 35) (375.0 ± 110.3 ng/ml vs. 357.7 ± 99.9 ng/ml, *p* = 0.482).

### 3.3. Correlation of Plasma CTRP3 Concentrations with the Other Parameters

Since there was statistical difference in plasma CTRP3 among patients with sinus rhythm and AF patients and the subgroups, we further analyzed the relationship between CTRP3 concentrations and clinical characteristics and laboratory findings. Pearson/Spearman correlation analysis revealed a negative correlation of plasma CTRP3 concentrations with AF (*r* = −0.285, *p* = 0.001), age (*r* = −0.315, *p* < 0.001), gender (*r* = −0.192, *p* = 0.034), Cr (*r* = −0.197, *p* = 0.030), and LAP (*r* = −0.237, *p* = 0.010) and a positive correlation of plasma CTRP3 concentrations with BMI (*r* = 0.190, *p* = 0.037) (Table 3). No linear relationship was found between the plasma CTRP3 concentrations and the remaining parameters, such as US-CRP, HbA1C, TC, TG, LDL-C, HDL, NT-pro BNP, LAD, LAA, and LVEF (*p* > 0.05).

### 3.4. Multivariate Logistic Regression Analysis of AF Rhythm and CTRP3 Concentration

To assess whether plasma CTRP3 was an independent predictor of AF, we included the variables with *p* < 0.05 in the univariate analysis in Table 1, such as age, NT-proBNP, LAD, LAA, LVEF, and CTRP3 and conducted multivariate logistic regression analysis. The result revealed that plasma CTRP3 concentrations and NT-proBNP were independent factors associated with AF, and plasma CTRP3 concentrations were negatively associated with AF (Table 4).

### 3.5. Relationship between Plasma CTRP3 Levels and (Short-Term 12 months) Prognosis of AF Ablation

**Figure 3:**
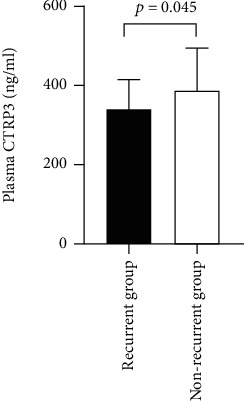
Relationship between plasma CTRP3 levels and (short-term 12 months) prognosis of AF ablation.

AF patients were followed up for 12 months. Excluding lost-to-follow-up patients, 71 AF patients completed the follow-up. During the follow-up period, 18 (25.4%) experienced recurrent AF after radiofrequency catheter ablation (RFCA). The level of plasma CTRP3 was significantly lower in the patients who experienced recurrence than that in those who did not have recurrence (337.9 ± 77.3 ng/ml vs. 386.6 ± 108.1 ng/ml, *p* = 0.045) (Figure 3).

## 4. Discussion

AF, one of the most common arrhythmia, is increasing the risk of stroke, disability, and morbidity. Studies have shown that classic adipokines such as adiponectin and leptin in plasma participate in the occurrence and development of AF through inflammatory response and oxidative stress [16, 17]. Besides, more and more investigators have found that epicardial adipose tissue may directly participate in atrial fibrosis and structural remodeling by secreting a variety of inflammatory factors and adipokines [18, 19]. CTRP3, a new type of adipokine, is a critical member of the C1q tumor necrosis factor-related proteins (CTRPs) family [20]. It is structurally similar to adiponectin [21, 22]. Adiponectin has been found to be involved in AF through inflammatory factors. Increased adiponectin levels have been found in atrial and peripheral adipose tissue under the stimulation of eicosapentaenoic acid in a rabbit heart failure model, accompanied by reduced tumor necrosis factor and decreased incidence of AF [23]. Adiponectin was also reported to be associated with atrial structural remodeling in human body. A study [19] had shown that adiponectin levels in AF patients were independently related to the adipose tissue and the thickness of atrial septum, which were also independently related to the volume of the left atrium. Therefore, it was speculated that adiponectin was related to atrial remodeling. Shimano et al. [24] found that plasma adiponectin level in patients with persistent AF were higher than those in paroxysmal AF and the control group, accompanied by an increase in plasma CITP (carboxy terminal telopeptide of collagen type I) levels. The correlation between adiponectin and CITP in patients with persistent AF suggested that adiponectin was involved in atrial remodeling, which was related to the occurrence and maintenance of atrial fibrillation. CTRP3, structurally similar to adiponectin, has been discovered to participate in myocardial remodeling with the functions of anti-inflammation and antiapoptosis [14]. Nevertheless, whether CTRP3 may be playing a role in atrial fibrillation and the concomitant atrial remodeling has not been reported.

Our study found that plasma CTRP3 concentrations were significantly lower in the AF group than in the control group. Further analysis discovered among the AF patients, persistent AF patients have lower plasma CTRP3 concentrations than paroxysmal AF patients. It was speculated that plasma CTRP3 might be involved in the development of AF. Multiple logistic regression analysis of the influencing factors of AF revealed that plasma CTRP3 was independently related to AF. The plasma CTRP3 level had nothing to do with the heart rhythm state for a short period since no significant difference was found between the patients with sinus rhythm and patients with AF when we compared the plasma CTRP3 concentrations in the AF patients according to the cardiac rhythm at admission. We can speculate that the relationship between CTRP3 level and atrial fibrillation is not determined by atrial contraction status with a short period of atrial rhythm, but the occurrence and development of atrial remodeling with a progressive process.

It seems that plasma CTRP3 is related to the occurrence of AF and the structural change of the atrium. We found an inverse correlation between CTRP3 level and left atrial pressure (LAP). The elevation of LAP also shortens the effective refractory period of cardiomyocytes and increases the chances of developing AF [25]. LAP was related to left atrial structural remodeling [26]. Some studies found that plasma CTRP3 levels were associated with hypertension, coronary heart disease, and heart failure, and CTRP3 inhibited myocardial fibrosis in myocardial infarction rats. In animal models of myocardial infarction (MI), exogenous CTRP3 pretreatment increases survival, improves postevent cardiac function, and prevents pathological remodeling [27]. CTRP3 can attenuate TGF-*β*1-induced signaling and pathogenic remodeling post-MI both in vivo and in vitro [28]. It significantly reduced myocardial interstitial fibrosis and reduced the expression of the inflammatory factor TGF-131 protein, suggesting that CTRP3 can decrease myocardial fibrosis and slow myocardial remodeling [29, 30]. Atrial fibrosis is the primary mechanism of atrial structural remodeling, and it has been verified by magnetic resonance imaging (MRI) [31]. The similarity between atrial fibrosis and ventricular fibrosis makes us speculate that plasma CTRP3 may participate in the development of AF by slowing atrial remodeling. In our study, plasma CTRP3 concentrations in the AF group were lower than that in the control group. We surmised that it was the reduction of this protective effect that led to atrial remodeling and led to atrial fibrillation, which may be one of the mechanisms. CTRP3 is structurally similar to adiponectin. But our study did find that the negative correlation between CTRP3 and AF was different from that of adiponectin and atrial fibrillation found in previous studies as they found positive correlation. This was really an interesting new discovery. We conjectured that there were many possible mechanisms, such as competitive inhibition, feedback regulation, and differences in receptor binding. However, the specific mechanism needs further design of animal models and experimental studies of molecular cell biology. Besides, CTRP3 is a potent anti-inflammatory mediator and is negatively associated with the proinflammatory cytokines TNF, interleukin-6 (IL-6), and C-reactive protein [32, 33]. More and more studies have proved that inflammation was involved in the occurrence and development of AF [31]. However, it is unknown whether CTRP3 is involved in the occurrence of AF and atrial remodeling through anti-inflammatory effect because our study did not find the correlation between CTRP3 and ultrasensitive CRP; further experiments are needed to elaborate the specific mechanism of CTRP3 involved in AF.

In this study, plasma CTRP3 level was significantly lower in the patients who experienced recurrence than in patients without recurrent AF. AF patients relapse after radiofrequency ablation is associated with many risk factors. Studies have shown that recurrence after RFCA was related to the size of the left atrium, inflammatory factors, type of atrial fibrillation, and metabolic syndrome. Therefore, we speculated that CTRP3 might participate in the recurrence of RFCA through atrial enlargement and atrial fibrosis. However, the study could only draw correlations, not causality. The specific mechanism needs further verification in vitro and animal experiments.

For the first time, the correlation between plasma CTRP3 and AF was reported. This study discovered the plasma CTRP3 of patients with AF was lower than that of the control group. It was speculated that CTRP3 was involved in the occurrence and development of AF. The underlying mechanism is still unclear, which will require further research.

## 5. Limitations

This study was a single-center, small-sample, nonpersistent observation study. There may be a certain degree of selection bias in the selection of patients. The small sample size also weakens the results. In order to be more persuasive, it needs to expand the sample size in future studies. This study only provides a correlation conclusion. It cannot explain the causal relationship between CTRP3 and AF. The exact mechanism of CTRP3 in AF needs further design of animal models and experimental studies of molecular cell biology.

## 6. Conclusion

The plasma CTRP3 concentrations in patients with AF were significantly lower than those in patients with sinus rhythm, and the plasma CTRP3 concentrations in patients with persistent AF were lower than those with paroxysmal AF. CTRP3 is an independent factor of AF, and to some extent, it is related to the prognosis of patients with AF after radiofrequency ablation.

## Figures and Tables

**Table 1:** Clinical and biochemical characteristics of AF patients and controls.

	AF patients (*n* = 75)	The controls (*n* = 47)	*p* value
Age (years)	62.5 ± 11.7	56.9 ± 11.4	0.011^∗^
Male *n* (%)	42 (56.0)	27 (57.4)	0.875
BMI (kg/m^2^)	25.3 ± 3.2	26.3 ± 3.9	0.098
SBP (mmHg)	130.9 ± 15.7	133.1 ± 17.8	0.496
DBP (mmHg)	77.4 ± 10.8	80.5 ± 16.1	0.207
Smoking *n* (%)	27 (36.0)	19 (40.4)	0.624
HT history *n* (%)	44 (58.7)	32 (68.1)	0.296
HLP history *n* (%)	41 (54.7)	33 (70.2)	0.087
DM history *n* (%)	13 (17.3)	14 (29.8)	0.107
CHD history *n* (%)	14 (18.9)	11 (23.4)	0.553
Anticoagulant *n* (%)	21 (28.0)	0 (0)	<0.001^∗^
Antiplatelet drugs *n* (%)	15 (20.0)	12 (25.5)	0.474
Statin *n* (%)	23 (30.7)	16 (34.0)	0.697
ACEI/ARB *n* (%)	16 (21.3)	17 (36.2)	0.073
CCB *n* (%)	25 (33.3)	18 (38.3)	0.576
*Β* blockers *n* (%)	31 (41.3)	12 (25.5)	0.075
HbAlc (%)	6.1 ± 1.0	6.4 ± 1.3	0.198
Cr (*μ*mol/l)	85.9 ± 12.5	86.5 ± 12.9	0.798
TC (mmol/l)	4.2 ± 0.9	4.2 ± 1.1	0.920
TG (mmol/l)	1.5 ± 0.8	1.7 ± 1.0	0.124
LDL-C (mmol/l)	2.6 ± 0.7	2.6 ± 1.0	0.878
HDL-C (mmol/l)	1.1 ± 0.3	1.0 ± 0.2	0.119
Us-CRP (mg/l)	0.76 (0.04, 23.98)	1.1 (0.10, 20.64)	0.131
NT-proBNP (pg/ml)	219.90 (16.86, 3584.00)	61.52 (6.43, 1046.00)	<0.001^∗^
LAD (mm)	36.7 ± 4.7	35.0 ± 4.1	0.041^∗^
LAA (cm^2^)	21.5 ± 4.2	18.7 ± 3.3	<0.001^∗^
LAP (mmHg)	11.3 ± 3.8	10.2 ± 3.5	0.122
LVEF (%)	67.8 ± 6.9	70.6 ± 4.4	0.009^∗^

NT-pro BNP and Us-CRP were presented as median (interquartile range); other characteristics were presented as means ± standard errors. The difference in gender and smoking was analyzed by chi-square tests; AF: atrial fibrillation; BMI: body mass index; SBP: systolic blood pressure; DBP: diastolic blood pressure; HT: hypertension; HLP: hyperlipidemia; DM: diabetes mellitus; CHD: coronary heart disease; ACEI: angiotensin-converting enzyme inhibitors; ARB: angiotensin receptor blocker; CCB: calcium channel blocker; HbAlc: hemoglobin A1c; Cr: creatinine; TC: total cholesterol; TG: triglycerides; LDL: low-density lipoprotein; HDL: high-density lipoprotein; Us-CRP: ultrasensitive C-reaction protein; NT-proBNP: N-Terminal probrain natriuretic peptide; LAD: left atrial diameter; LAA: left atrial area; LAP: left atrial pressure; LVEF: left ventricular ejection fraction. ^∗^*p* < 0.05.

**Table 2:** Clinical and biochemical characteristics of AF subgroups.

	Paroxysmal AF (*n* = 56)	Persistent AF (*n* = 19)	*p* value
Age (years)	63.0 ± 11.8	61.1 ± 11.9	0.543
Gender male *n* (%)	26 (46.4)	16 (84.2)	0.004∗
BMI (kg/m^2^)	25.0 ± 3.4	26.1 ± 2.6	0.185
SBP (mmHg)	131.8 ± 15.8	128.6 ± 15.5	0.446
DBP (mmHg)	76.4 ± 10.9	80.5 ± 10.0	0.148
Smoking *n* (%)	19 (33.9)	8 (42.1)	0.521
HT history *n* (%)	33 (58.9)	11 (57.9)	0.937
HLP history *n* (%)	31 (55.4)	10 (52.6)	0.837
DM history *n* (%)	9 (16.1)	4 (21.1)	0.885
CHD history *n* (%)	11 (20.0)	3 (15.8)	0.949
Anticoagulant *n* (%)	14 (25.0)	7 (36.8)	0.321
Antiplatelet drugs *n* (%)	12 (21.4)	3 (15.8)	0.842
Statin *n* (%)	19 (33.9)	4 (21.1)	0.445
ACEI/ARB *n* (%)	12 (21.4)	4 (21.1)	1.000
CCB *n* (%)	20 (35.7)	5 (26.3)	0.453
*Β* blockers *n* (%)	20 (35.7)	11 (57.9)	0.090
HbAlc (%)	6.0 ± 0.6	6.5 ± 1.5	0.113
Cr (*μ*mol/l)	83.7 ± 12.3	92.5 ± 10.7	0.007^∗^
TC (mmol/l)	4.3 ± 0.9	4.2 ± 1.0	0.699
TG (mmol/l)	1.5 ± 0.9	1.4 ± 0.6	0.706
LDL-C (mmol/l)	2.6 ± 0.7	2.7 ± 0.8	0.550
HDL-C (mmol/l)	1.1 ± 0.3	1.0 ± 0.2	0.090
Us-CRP (mg/l)	0.78 (0.10, 23.98)	0.64 (0.04, 6.21)	0.946
NT-proBNP (pg/ml)	144.6 (6.43, 3584.0)	543.3 (160.80, 3391.00)	<0.001^∗^
LAD (mm)	35.8 ± 4.2	39.4 ± 5.0	0.003^∗^
LAA (cm^2^)	20.3 ± 3.3	25.0 ± 4.4	<0.001^∗^
LAP (mmHg)	11.1 ± 3.3	11.8 ± 5.0	0.568
LVEF (%)	68.7 ± 6.3	65.4 ± 7.9	0.070

NT-pro BNP and Us-CRP were presented as median (interquartile range); other characteristics were presented as means ± standard errors. The difference in gender and smoking was analyzed by chi-square tests; AF: atrial fibrillation; BMI: body mass index; SBP: systolic blood pressure; DBP: diastolic blood pressure; HT: hypertension; HLP: hyperlipidemia; DM: diabetes mellitus; CHD: coronary heart disease; ACEI: angiotensin-converting enzyme inhibitors; ARB: angiotensin receptor blocker; CCB: calcium channel blocker; HbAlc: hemoglobin A1c; Cr: creatinine; TC: total cholesterol; TG: triglycerides; LDL: low-density lipoprotein; HDL: high-density lipoprotein; Us-CRP: ultrasensitive C-reaction protein; NT-proBNP: N-Terminal probrain natriuretic peptide; LAD: left atrial diameter; LAA: left atrial area; LAP: left atrial pressure; LVEF: left ventricular ejection fraction. ^∗^*p* < 0.05.

**Table 3:** Relationship between CTRP3 and other variables.

	Correlation coefficient (*r*)	*p* value
AF	-0.285	0.001^∗^
Age	-0.315	<0.001^∗^
Gender	-0.192	0.034^∗^
BMI	0.190	0.037^∗^
Cr	-0.197	0.030^∗^
LAP	-0.237	0.010^∗^

AF: atrial fibrillation; BMI: body mass index; Cr: creatinine; LAP: left atrial pressure. ^∗^Statistically significant at two-sided *α* = 0.05 level.

**Table 4:** Logistic regression analysis for the presence of AF.

	*B*	S.E.	OR	95% CI	*p*
Gender	-0.366	0.565	0.693	0.229-2.097	0.517
Age	0.007	0.025	1.007	0.958-1.057	0.793
BMI	-0.103	0.092	0.902	0.752-1.081	0.263
CHD	-0.672	0.678	0.511	0.135-1.928	0.322
HT	-1.045	0.553	0.352	0.119-1.039	0.059
DM	-0.752	0.663	0.471	0.129-1.727	0.256
NT-proBNP	0.004	0.002	1.004	1.000-1.007	0.042∗
LAD	-0.006	0.095	0.994	0.826-1.197	0.951
LAA	0.197	0.116	1.218	0.970-1.530	0.090
LVEF	-0.079	0.049	0.924	0.839-1.018	0.112
CTRP3	-0.005	0.003	0.995	0.989-1.000	0.048∗

BMI: body mass index; HT: hypertension; DM: diabetes mellitus; CHD: coronary heart disease; NT-proBNP: N-Terminal probrain natriuretic peptide; LAD: left atrial diameter; LAA: left atrial area; LVEF: left ventricular ejection fraction. ^∗^Statistically significant at two-sided *α* = 0.05 level.

## Data Availability

The excel data used to support the findings of this study are available from the corresponding author upon request.

## Abbreviations

AF: Atrial fibrillation
CTRP3: Complement C1q tumor necrosis factor-related protein 3
ELISA: Enzyme-linked immunosorbent assay
AST: Aspartate aminotransferase
ALT: Alanine aminotransferase
BMI: Body mass index
US-CRP: Ultrasensitive C-reactive protein
Cr: Creatinine
HbA1C: Hemoglobin A1C
TC: Total cholesterol
TG: Triglycerides
LDL-C: Low-density lipoprotein cholesterol
HDL-C: High-density lipoprotein cholesterol
NT-proBNP: N-terminal probrain natriuretic peptide
LAA: Left atrial area
LAD: Left atrial diameter
LAP: Left atrial pressure
LVEF: Left ventricular ejection fraction
ECG: Electrocardiograph
RFCA: Radiofrequency catheter ablation
CITP: Carboxy terminal telopeptide of collagen type I
MI: Myocardial infarction
TGF: Transforming growth factor
MRI: Magnetic resonance imaging
TNF: Tumor necrosis factor
IL-6: Interleukin-6.
